# Supplementary material for: Content-rich biological network constructed by mining PubMed abstracts
Source: BMC Bioinformatics. 2004 Oct 8;5:147. doi: 10.1186/1471-2105-5-147 (PMC528731; doi:10.1186/1471-2105-5-147)
Supplement: Additional File 2 — The original results of the above study (non-essential files are deleted to keep the file size under the limit set by BMC bioinformatics). [file 1471-2105-5-147-S2.bz2 › chilibotAdditionalFile2/dip05/314ID9013879E1371/html/IL13RA1_IL4R.html]

 


 **IL13RA1** and **IL4R** 
  
Found 5 abstracts in PubMed, retrieved 05.  
 

 What does Google say? 
 PDF only 
| .edu only 

---

**Interactive relationship** (e.g. stimulation, inhibition, etc)

**Neutral relationship**- The investigation of the subunit structure of IL 4R demonstrated that head and neck cancer cell lines expressed mRNA for IL 4R beta chain also known as IL 4R alpha  [ **IL4R** ]  and IL 13R alpha chain also known as IL 13R alpha1  [ **IL13RA1** ] .  Ref: 10850446 Cancer Res, 2000

**Non-interactive relationship** (e.g. studied together, co-existance, homology, etc.)

- In fact, IL 4R alpha  [ **IL4R** ]  with IL 13R alpha1  [ **IL13RA1** ]  is designated both as an IL 13 receptor and the type II IL 4 receptor.  Ref: 10534111 J Leukoc Biol, 1999
- Cloning of the human IL 13R alpha1  [ **IL13RA1** ]  chain and reconstitution with the  **IL4R**  alpha of a functional IL 4 IL 13 receptor complex.  Ref: 9013879 FEBS Lett, 1997
- We hypothesized that variants in the IL13,  **IL13RA1** , and IL4RA  [ **IL4R** ]  genes would be associated with an accelerated rate of decline of lung function among smokers.  Ref: 12594065 Am J Respir Cell Mol Biol, 2003
- We determined the allele frequencies of five polymorphisms in the IL13,  **IL13RA1** , and IL4RA  [ **IL4R** ]  genes in 588 continuing smokers chosen from the NHLBI Lung Health Study for having the fastest n = 282 and slowest n = 306 5 yr rate of decline of lung function mean change in FEV 1 %predicted yr = 4.1 and 1.1, respectively.  Ref: 12594065 Am J Respir Cell Mol Biol, 2003
- Polymorphisms in the IL13,  **IL13RA1** , and IL4RA  [ **IL4R** ]  genes and rate of decline in lung function in smokers.  Ref: 12594065 Am J Respir Cell Mol Biol, 2003
- Electrophoretic mobility shift assay showed that IL 13 and IL 4 were able to activate Stat6 in cells expressing both IL 4R alpha  [ **IL4R** ]  and IL 13R alpha1  [ **IL13RA1** ] , while no activation was observed in cells expressing either one or the other alone.  Ref: 9013879 FEBS Lett, 1997
